# A parametric model to jointly characterize rate, duration, and severity of exacerbations in episodic diseases

**DOI:** 10.1186/s12911-022-02080-5

**Published:** 2023-01-12

**Authors:** Abdollah Safari, John Petkau, Mark J. FitzGerald, Mohsen Sadatsafavi

**Affiliations:** 1 Department of Mathematics, Statistics, and Computer Science, University of Tehran, Tehran, Iran; 2 Respiratory Evaluation Sciences Program, Collaboration for Outcomes Research and Evaluation, Faculty of Pharmaceutical Sciences, University of British Columbia, Vancouver, Canada; 3 Department of Statistics, University of British Columbia, Vancouver, Canada; 4 Centre for Lung Health, Vancouver Coastal Health Research Institute, Vancouver, Canada

**Keywords:** Recurrent episodes, Recurrent events, Random effect models, Gap times, Asthma exacerbations, Time-to-event analysis

## Abstract

**Background:**

The natural history of many chronic diseases is characterized by periods of increased disease activity, commonly referred to as flare-ups or exacerbations. Accurate characterization of the burden of these exacerbations is an important research objective.

**Methods:**

The purpose of this work was to develop a statistical framework for nuanced characterization of the three main features of exacerbations: their rate, duration, and severity, with interrelationships among these features being a particular focus. We jointly specified a zero-inflated accelerated failure time regression model for the rate, an accelerated failure time regression model for the duration, and a logistic regression model for the severity of exacerbations. Random effects were incorporated into each component to capture heterogeneity beyond the variability attributable to observed characteristics, and to describe the interrelationships among these components.

**Results:**

We used pooled data from two clinical trials in asthma as an exemplary application to illustrate the utility of the joint modeling approach. The model fit clearly indicated the presence of heterogeneity in all three components. A novel finding was that the new therapy reduced not just the rate but also the duration of exacerbations, but did not have a significant impact on their severity. After controlling for covariates, exacerbations among more frequent exacerbators tended to be shorter and less likely to be severe.

**Conclusions:**

We conclude that a joint modeling framework, programmable in available software, can provide novel insights about how the rate, duration, and severity of episodic events interrelate, and enables consistent inference on the effect of treatments on different disease outcomes.

*Trial registration* Ethics approval was obtained from the University of British Columbia Human Ethics Board (H17-00938).

**Supplementary Information:**

The online version contains supplementary material available at 10.1186/s12911-022-02080-5.

## Background

The course of many chronic diseases is highlighted by periodic worsening, commonly referred to as exacerbations, flare-ups, or attacks. Examples include asthma, chronic obstructive pulmonary disease (COPD), multiple sclerosis, Parkinson’s disease, and cystic fibrosis. These exacerbations are often an important component of the natural history of such diseases and constitute a major source of their burden. Given the substantial contribution of exacerbations to the overall burden of such diseases, characterizing the occurrence and intensity of exacerbations is a major focus of research and patient care [1, 2].

In general, the total burden of exacerbations is determined by three dimensions: their rate (frequency), their duration, and their severity. Individual patients might differ in the extent of these dimensions. For example, in both asthma and COPD, it has been established that individuals have significantly different rates of exacerbation [3–5], as well as different tendencies towards experiencing severe versus mild exacerbations [6]. Further, as interventions that target exacerbations can differentially affect each dimension, a comprehensive understanding of treatment effect requires evaluating all three dimensions simultaneously.

Evaluating interventions that target the burden of exacerbations has conventionally focused on exacerbation rate. This is manifested in exacerbation rate (or risk) being the primary end-point in the majority of clinical trials in asthma. Common analytic approaches include marginal models [7–9], modeling time to the first exacerbation in a survival analysis framework [10], using models for count outcomes [11, 12], or employing models for recurrent events [13, 14]. Recent developments in the analysis of exacerbation trials include the use of random effect models to account for between-individual variability in exacerbation rates [13, 14], and the use of a mixture of a random effect or a gap-time model with a logistic regression model to allow for the excessive presence of individuals without any exacerbations (“zero inflation”) during follow-up [13–15]. Another approach could be using marginal regression analysis of recurrent point processes (and their extensions) for recurrent events (see, e.g., [16]). See [17] as a recent systematic review of different models for recurrent events data.

The commonality among these developments is the desire for more accurate modeling of exacerbation rate. On the other hand, the majority of such models consider exacerbations as instantaneous events. Ignoring exacerbation duration may lead to biased rate estimates [18]. To address this issue, [19] extended the Cox proportional hazards model for situations where the event is recurrent and the event duration is non-negligible. They did not explicitly model the durations, but adjusted the risk set to accommodate event duration in order to improve modeling of event rate. To enable inference on both event rate and duration, [20, Chapter 6] proposed an alternating two-state process, which paralleled the two states of “at risk” and “not at risk”, and modeled the event rate and duration using the times of transitions between the two states. Very few previous studies have explored how the severity of exacerbations can be studied in tandem with their frequency. An exception is a recently proposed joint frailty-logistic model for simultaneous inference on exacerbation rate and severity [6, 21]; however, exacerbation duration was not evaluated in this framework.

**Fig. 1:**
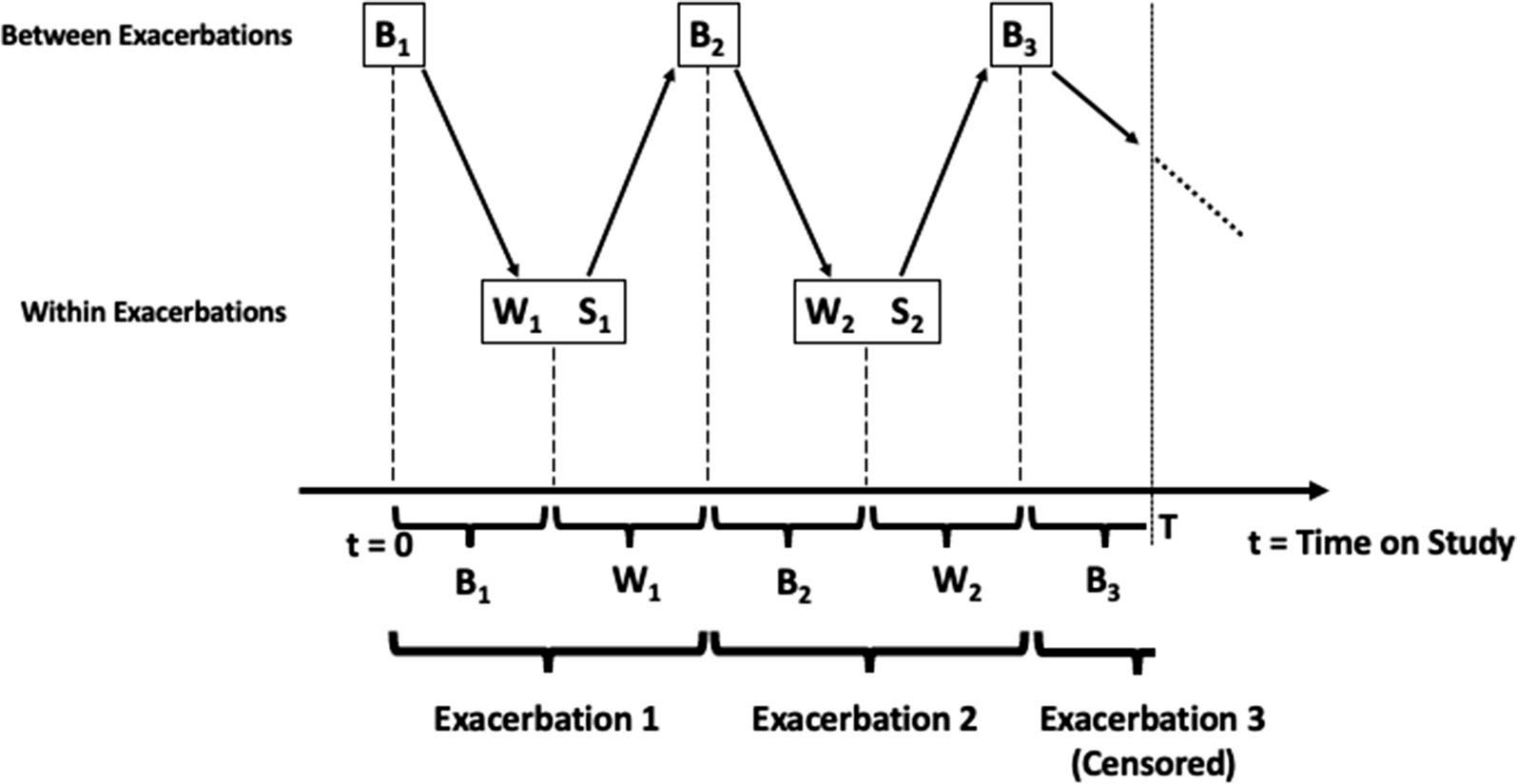
Schematic illustration of model specification. This graph illustrates within (*W*’s) and between (*B*’s) exacerbation durations (gap times), exacerbation severity (*S*’s), and follow-up time termination (*T*) for a subject who had two exacerbations during the follow-up period

The purpose of this work is to expand on previous developments to provide a unified inferential framework for all three dimensions of exacerbations. Such a unified framework has two important potentials. First, it can provide better insight into the natural history of exacerbations; for example, this framework enables one to assess the hypothesis that individuals who exacerbate more frequently are generally more susceptible to the causes of such events, and concordantly experience longer and more severe events. Second, this framework enables more comprehensive evaluation of treatment effects, by enabling simultaneous inference on determinants of the burden of exacerbations and not just rate. The implementation of our framework is provided in available commercial software, facilitating access for the applied research community.

## Motivation: a case study in asthma

As an example of a chronic disease with episodic events, we studied asthma where exacerbations are a source of considerable morbidity for patients and economic burden for both patients and providers [22, 23]. We used pooled data from two large randomized placebo-controlled trials (DREAM and MENSA [24, 25]) of the new biologic medication mepolizumab to illustrate how our unified framework is able to comprehensively characterize the burden of exacerbations and the impact of this novel therapy on the different aspects of this burden.

Briefly, in DREAM, 621 patients were randomized to placebo or one of three doses of intravenous mepolizumab (75 mg, 250 mg, or 750 mg), and were followed for 52 weeks. In MENSA, 580 patients were randomized to placebo or one of two doses of mepolizumab (75 mg or 100 mg), and were followed for 32 weeks. Preliminary analyses (involving descriptive analysis of patient characteristics and observed outcomes, and fitting the model with study ID and its interaction with treatment arms) supported the pooling of the data from the two trials (see Section 1 of the Additional file 1 for more details). The pooled data therefore provided outcomes for placebo and four different doses of mepolizumab.

The primary outcome in both studies was the rate of asthma exacerbations. Exacerbations were defined consistently in both studies. The timing since randomization, the duration, and severity of exacerbations were recorded. For the purposes of our study, we consider exacerbation severity as a binary response: exacerbations requiring an emergency department visit or hospital admission are considered severe, while those managed in outpatient settings are considered not severe. All individuals were exacerbation-free at the beginning of follow-up.

## Methods

All methods were carried out in accordance with relevant guidelines and regulations.

### Definitions and notation

Let states 1 and 2 represent the recovered and exacerbation states, respectively. Suppose *n* patients were followed over time to generate data on *n* independent alternating two-state processes. For patient *i* ($i = 1, \ldots , n$), let $M_i$ be the number of observed exacerbations over their follow-up time $T_i$. In our case study, the times $T_i$ differ across patients because the nominal follow-up periods differed in the two trials under consideration, some patients dropped out during follow-up, and in both studies patients who were experiencing an exacerbation at the end of their nominal follow-up period were further followed to the termination of that event.

**Fig. 2:**
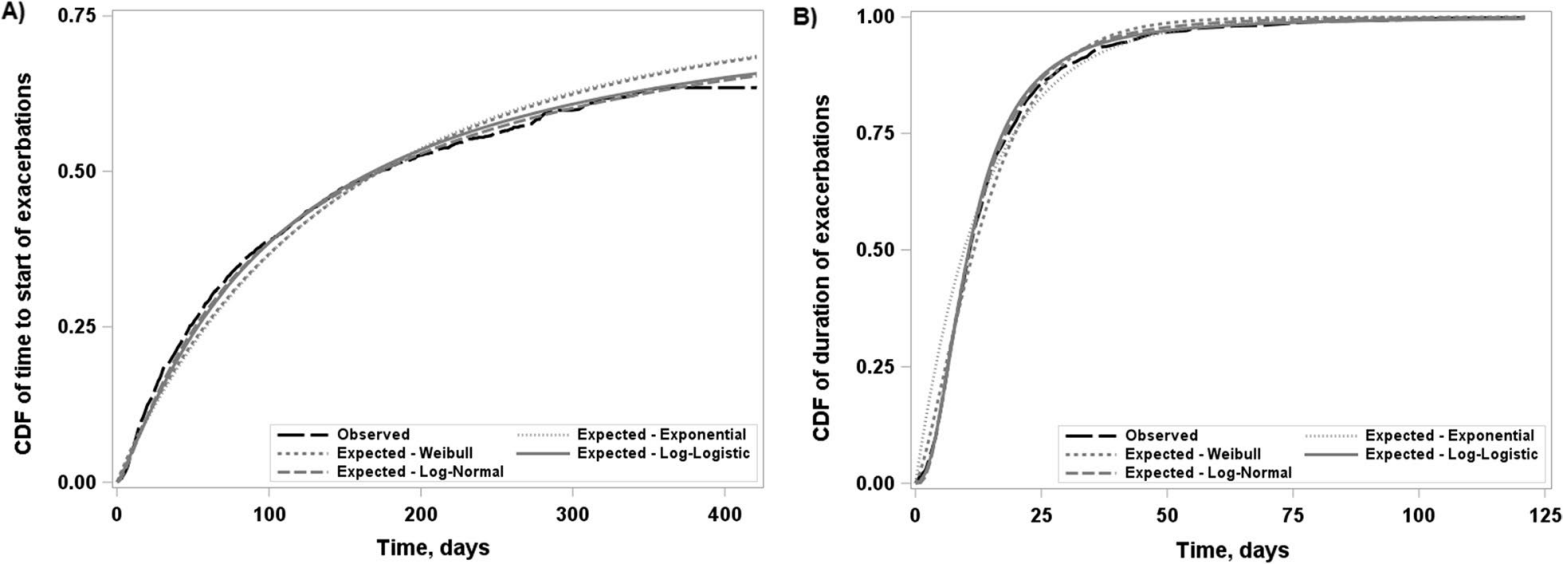
Empirical (observed) versus estimated marginal cumulative distribution functions (averaged over the predictors) of between-exacerbation (**A**) and within-exacerbation (**B**) times

For the *j*th exacerbation of the *i*th patient, let $U_{i,j}$ and $V_{i,j}$ be, respectively, the time of exacerbation onset and termination. Thus, the exacerbation duration (within-exacerbation period) is $W_{i,j}=V_{i,j}-U_{i,j}$ and the between-exacerbation period (the time from the termination of the previous exacerbation to the beginning of the *j*th exacerbation) is $B_{i,j}=U_{i,j}-V_{i,j-1}$. In the recurrent event literature, these periods are often referred to as gap times. Since we do not know the termination time of the patient’s last exacerbation prior to study entry, we assume the randomization date coincides with the beginning of the initial exacerbation-free period; that is, we set $V_{i,0} \equiv 0$. We will later discuss the implications of this simplifying assumption in the context of our case study. Similarly, we define $S_{i,j}$ as the binary severity outcome of the *j*th exacerbation ($S_{i,j}=1$ if the exacerbation is severe, $S_{i,j}=0$ otherwise). We assume the censoring due to dropout occurs independently of the within- and between-exacerbation gap times and severity. In our case study, only the between-exacerbation gap times are censored as all individuals who were experiencing an exacerbation at the end of the nominal follow-up period were followed until the end of that exacerbation. Extension of our approach for situations with censored within-exacerbation gap times is immediate. Figure 1 provides a schematic illustration of the history of exacerbations in a given patient and the relationship between the three disease features of interest.

In addition, we let $\varvec{X}_{i,j}$ (for $j = 1, \ldots ,M_i$) be the vector of covariates for the *j*th episode of patient *i*; the vector $\varvec{X}_{i,j}$ includes both baseline covariates and episode-specific covariates that are measured at the onset of the *j*th exacerbation-free period. For simplicity of notation, in what follows we consider the same set of covariates $\varvec{X}_{i,j}$ for modeling each of the three features, but optionally different sets of covariates can be used for each component.

### The model

We propose a parametric model to jointly characterize the distribution of $B_{i,j}$ (between-exacerbation gap time, related to exacerbation rate), $W_{i,j}$ (within exacerbation gap time or duration), and $S_{i,j}$ (severity of exacerbation). Each of the three submodels incorporates a random effect to capture heterogeneity beyond that explained by included covariates, thus inducing autocorrelations among the repeated gap times of each type and the repeated severities within a patient. Joint modeling of the three random effects for each patient also allows for inter-correlations among the three model dimensions. We opted for parametric specification of all model components to allow straightforward estimation of random effects and prediction of outcomes. A further advantage of a parametric specification is that the likelihood function for the model can readily be programmed in available software.

#### The rate submodel

We use hazard-based models for the alternating two-state process [20]. The event hazard function gives the instantaneous rate of an event occurring at time *t*, conditional on the process history. For simplicity, we suppress the dependence on the process history and covariates in our notation. For the onset of an exacerbation event (the between-exacerbation gap times $B_{i,j}$), we use an accelerated failure time (AFT) model with an additive random effect (RE) [26, 27]. Specifically, conditional on a zero-mean individual-specific random effect $Z_{B,i}$, the $B_{i,j}$’s of patient *i* ($i=1, \ldots ,n$ and $j=1, \ldots ,M_i$; for simplicity, the range of the indices will be suppressed hereafter) are assumed to be independent. The hazard function for $B_{i,j}$ is modelled as

$$\begin{aligned} h_{i,j,B} \left( t | Z_{B,i} \right) =\theta _{i,j,B} h_B^0 \left( \theta _{i,j,B} \left( t-V_{i,j-1} \right) \right),\quad for\quad t > V_{i,j-1} \end{aligned}$$

where $h_B^0 (.)$ is the baseline hazard function for the onset of exacerbation events and $\theta _{i,j,B}=\exp \left( -\eta _{i,j,B} \right)$ where $\eta _{i,j,B}=\varvec{X}_{i,j}^{T} \varvec{\beta }_B+ Z_{B,i}$, with $\varvec{\beta }_B$ the covariate coefficient vector.

This parametrization of the AFT, which can equivalently be represented as

$$\begin{aligned} \log \left( B_{i,j} \right) = \eta _{i,j,B} + \epsilon _{i,j,B} \end{aligned}$$

where $\exp \left( \epsilon _{i,j,B} \right)$ has hazard function $h_B^0 (.)$, has the attractive feature that interpretations of the regression coefficients are the same either conditionally (on the random effect) or marginally [28]. We refer to this as an AFT-RE model.

**Fig. 3:**
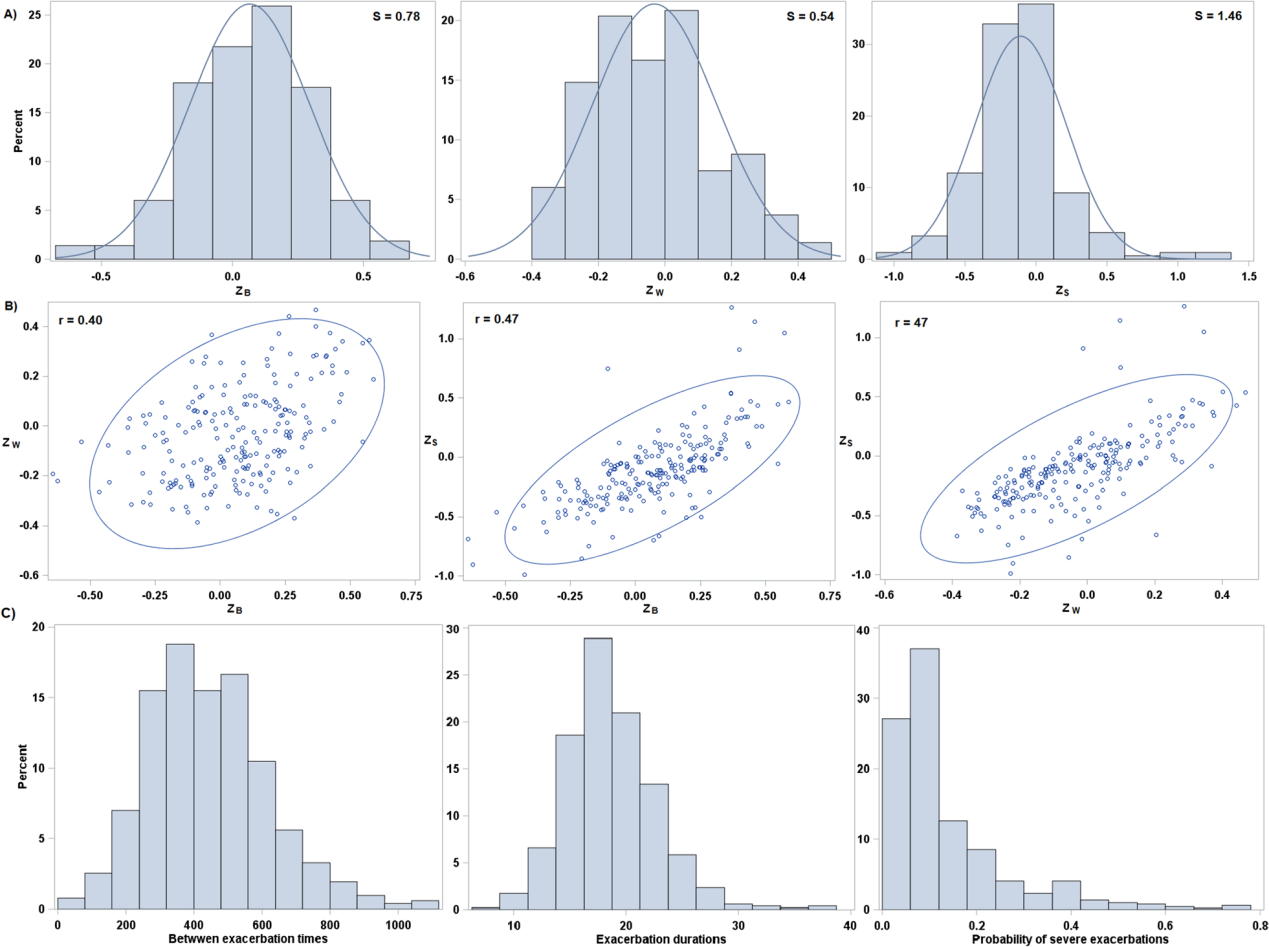
Histograms of estimated random effects with superimposed approximating normal densities (**A**), pairwise scatterplots of estimated random effects with 95% confidence ellipses (**B**), and histograms of marginal predicted mean outcomes (**C**). Estimated random effects ($\widehat{Z}_{B,i}$, $\widehat{Z}_{W,i}$, and $\widehat{Z}_{S,i}$) are obtained using the empirical Bayes method, implemented with the PREDICT statement of SAS PROC NLMIXED. As the random effect estimates of the duration and severity submodels are meaningful only for patients with at least one exacerbation during the follow-up period, only such patients are included in these plots

Parametric time to event models require specification of the baseline hazard function. We suggest evaluating different functions and making a selection based on goodness-of-fit (e.g., as measured by the Akaike Information Criterion [AIC] [29]), as well as visual comparison of observed and predicted time to event curves. In contrast to hazard ratios estimated from proportional hazards models, the regression coefficients of AFT models can be interpreted intuitively and simply in terms of factors that contribute to the acceleration or deceleration of the time to events [30]. More precisely, with the parametrization of the AFT hazard function specified above, if $\beta$ is the regression coefficient for a particular covariate, a one unit increase in that covariate is associated with multiplication of the expected time to event by the factor $\exp (\beta )$. In what follows, we refer to these as AFT factors.

#### The duration submodel

We use another AFT-RE model for the termination of an exacerbation event (the within-exacerbation gap times $W_{i,j}$). Conditional on a zero-mean individual-specific random effect $Z_{W,i}$, the $W_{i,j}$’s of patient *i* are assumed to be independent. The hazard function for $W_{i,j}$ is modelled as

$$\begin{aligned} h_{i,j,W} \left( t | Z_{W,i} \right) = \theta _{i,j,W} h_W^0 \left( \theta _{i,j,W} \left( t-U_{i,j} \right) \right)\quad for\quad t > U_{i,j} \end{aligned}$$

where $h_W^0 (.)$ is the baseline hazard function for the termination of exacerbation events and $\theta _{i,j,W} = \exp \left( -\eta _{i,j,W} \right)$ where $\eta _{i,j,W} = \varvec{X}_{i,j}^{T} \varvec{\beta }_W+ Z_{W,i}$, with $\varvec{\beta }_W$ the covariate coefficient vector. This parametrization of the AFT can equivalently be represented as

$$\begin{aligned} \log \left( W_{i,j} \right) = \eta _{i,j,W} + \epsilon _{i,j,W} \end{aligned}$$

where $\exp \left( \epsilon _{i,j,W} \right)$ has hazard function $h_W^0 (.)$. Again, one can evaluate several parametric baseline hazard functions and make a selection based on goodness-of-fit.

#### The severity submodel

We specify a logistic regression model for the conditional probability of an exacerbation being severe once it occurs, $p_{i,j} = P \left( S_{i,j} = 1 | Z_{S,i} \right)$, as

$$\begin{aligned} logit \left( p_{i,j} \right) = \varvec{X}_{i,j}^{T} \varvec{\beta }_S + Z_{S,i} \end{aligned}$$

where $\varvec{\beta }_S$ is the covariate coefficient vector. Conditional on a zero-mean individual-specific random effect $Z_{S,i}$, the $S_{i,j}$’s of patient *i* are assumed to be independent.

#### The zero-inflated component

In many circumstances, there is an excess number of patients who experience no exacerbations over the follow-up period (either because they are not susceptible to exacerbations or have a very low rate of events - a so-called “ non-susceptible” subgroup). This is similar to the situation motivating ‘cure models’ in survival analysis to model the presence of long-term survivors [31, 32]. To accommodate this aspect of the population, one can consider a mixture component which models $\pi _i$, the probability patient *i* is non-susceptible, through a logistic regression model in terms of the baseline covariates $\varvec{X}_{i,0}$ as

$$\begin{aligned} logit ( \pi _i ) = \varvec{X}_{i,0}^{T} \varvec{\beta }_{ZI} \end{aligned}$$

where $\varvec{\beta }_{ZI}$ is the covariate coefficient vector. As there are no repeated events related to this mixture modeling, we do not include a RE in this model component.

#### Modeling the interdependencies

We assume for patient *i*, given the random effects, $B_{i,j}$ and ($W_{i,j},S_{i,j}$) are independent, and given the random effects and $B_{i,j}$, $W_{i,j}$ and $S_{i,j}$ are independent (for $j=1, \ldots , M_i$). These assumptions allow for two types of correlation between the outcomes based on the random effects. The random effects in each submodel account for autocorrelation in that outcome across repeated events on the same patient. In addition, to accommodate potential dependencies among the three outcomes (e.g., if individuals with higher exacerbation rates tend to experience more severe exacerbations), we allow for correlation between the random effects across submodels. Specifically, we assume $\varvec{Z}_i = (Z_{B,i}, Z_{W,i}, Z_{S,i} ) ^ T$, $i=1, \ldots , n$, are independent and identically distributed with mean zero. For our motivating case study, we will take this distribution to be multivariate normal with covariance matrix $\Sigma _{\varvec{Z}}$. The distribution of the random effects is thus governed by 3 variance and 3 correlation parameters; these correlation parameters indirectly describe the relationships across patients between the rate, duration, and severity of the exacerbations.

### Implementation

The likelihood function is provided in Section 2 of the Additional file 1. We use PROC NLMIXED in SAS (with the built-in Newton–Raphson ridge optimization algorithm) to obtain the maximizer of the full likelihood, together with its estimated variance-covariance matrix (using SAS 9.4 PROC NLMIXED [SAS Institute, Cary NC]). To obtain the AFT factors/odds ratios (ORs) and their confidence intervals (CIs), we exponentiate the estimated coefficients and their CIs from the linear predictors. An annotated SAS macro that implements the model in a generic fashion, along with a manual and a simulated dataset, is available at our website (http://resp.core.ubc.ca/software/RDSmodel). The macro flexibly accommodates various aspects of the model such as the inclusion or exclusion of the duration and severity submodels, or which submodels should include a random effect.

## Results

### Characteristics of patients

Table 1 provides the characteristics of the final sample, including the covariates and outcomes. After excluding 84 and 7 patients because of missing or outlying covariate values, respectively, 1,110 patients remained in the final dataset (mean age 49.2, SD=12.9, 60.0% female). We conducted a sensitivity analysis to assess the impact of removing patients with missing values in covariates (Additional file 1—Section 3). The average follow-up time was 0.78 years and the overall withdrawal rate was 11%, with similar rates across all trial arms. As there were only 4 deaths and none of these were related to the disease, we did not incorporate death as a competing risk event in the model. These patients experienced 1,128 exacerbations (with an average duration of 14.4 days), corresponding to an exacerbation rate of 1.31 per year. The largest number of exacerbations observed in a patient was 10, but 52.8% of the patients did not experience any events during the study period. Of the exacerbations, 181 (16.0%) were severe.

After consulting with clinical experts, we included fourteen baseline covariates (listed in Table 1) in all four submodels. Two episode-specific covariates were also utilized: the number of previous exacerbations during follow-up (*N*) and the number of previous severe exacerbations during follow-up ($N_S$).

**Table 1:** Descriptors for baseline characteristics (average (SD) or count (%), as appropriate) and outcomes of the final sample

Predictors	Overall (*n*=1110)	Placebo* (*n*=323)	75 mg Mepolizumab* (*n*=317)	100 mg Mepolizumab (*n*=171)	250 mg Mepolizumab (*n*=150)	750 mg Mepolizumab (*n*=149)
Female	668 (60%)	193 (60%)	193 (61%)	102 (60%)	92 (61%)	88 (59%)
Age (years)	49.0 (12.9)	47.7 (13.2)	49.8 (13.0)	50.3 (14.5)	49.1 (11.5)	48.3 (11.1)
Body Mass Index (kg/m${}^2$)	28.1 (5.9)	28.2 (5.8)	28.0 (5.9)	27.7 (6.0)	28.4 (5.9)	28.7 (5.8)
Duration of asthma (years)	19.3 (14.0)	18.8 (14.3)	19.3 (13.9)	20.2 (12.9)	20.4 (14.0)	18.7 (15.0)
Maintenance daily dose of oral corticosteroids (mg)	4.2 (10.3)	3.5 (8.2)	3.7 (8.9)	3.4 (8.3)	6.4 (17.0)	5.1 (10.3)
Nasal polyps	147 (13%)	47 (14%)	40 (13%)	25 (15%)	22 (15%)	13 (9%)
Percentage of predicted pre-bronchodilator FEV1	60.3 (17.1)	61.1 (16.9)	60.2 (17.5)	59.7 (17.7)	59.3 (16.8)	60.7 (16.0)
FEV1 reversibility (%)	26.4 (21.6)	26.7 (22.4)	26.2 (20.2)	28.9 (25.1)	26.4 (21.0)	23.4 (18.9)
Score on asthma control questionnaire	2.3 (1.1)	2.3 (1.3)	2.2 (1.1)	2.2 (1.2)	2.4 (1.1)	2.2 (1.1)
Blood eosinophil count ($10^9/$L)	0.42 (0.40)	0.44 (0.42)	0.40 (0.38)	0.45 (0.43)	0.39 (0.44)	0.36 (0.31)
IgE (U/ml)	447 (1093)	435 (850)	548 (1318)	354 (364)	361 (847)	450 (1593)
Ethnicity (Black/Hispanic)	107 (10%)	31 (10%)	32 (10 %)	16 (9%)	14 (9%)	14 (9%)
History of smoking	247 (22%)	67 (21%)	74 (24%)	41 (24%)	30 (20%)	35 (24%)
Exacerbations requiring admission in year prior to study	3.6 (2.9)	3.7 (3.3)	3.6 (2.7)	3.8 (2.8)	3.4 (2.4)	3.5 (2.9)
Follow-up time (years)	0.78 (0.24)	0.75 (0.24)	0.75 (0.24)	0.61 (0.11)	0.94 (0.21)	0.92 (0.22)
Outcomes						
Number of exacerbations (annual rate)	1128 (1.31)	468 (1.94)	251 (1.05)	86 (0.83)	179 (1.27)	144 (1.04)
Duration of exacerbations (days)	14.4 (11.6)	15.0 (12.6)	15.1 (13.3)	12.9 (9.5)	14.1 (8.5)	12.4 (10.0)
Number of severe exacerbations (annual rate)	181 (0.21)	68 (0.28)	39 (0.16)	14 (0.13)	32 (0.23)	28 (0.20)

*Pooled data from both studies FEV1: Forced expiratory volume at one second

### Model outputs

We examined exponential, Weibull, log-normal, and log-logistic baseline hazard functions. As our primary purpose with the case study was to illustrate the utility of the modeling approach, for ease of presentation, attention was focused on use of the same distribution for both the between- (rate) and within- (duration) exacerbation gap times ($B_{i,j}$ and $W_{i,j}$). As judged in terms of AIC (Additional file 1—Section 3) and visual agreement between the observed and estimated marginal survival curves (Fig. 2), the log-normal provided the best fit. Not surprisingly, as more than half of patients did not experience any exacerbations during the study period, the zero-inflated component yielded a substantial reduction in the AIC and hence was retained in the final model.

The estimated baseline hazard functions for both rate and duration were nearly constant, so the different distributions provided a similar fit to that of the exponential as shown in Fig. 2. It follows that the baseline hazard function for the rate did not depend in any substantial way on the time since the termination the previous exacerbation. This is reassuring as it indicates negligible impact of the assumption that the first between-exacerbation gap time starts from the randomization date, while in fact this time is left-truncated.

Table 2 reports the estimated AFT factors in the rate and duration submodels, and the estimated ORs in the logistic regression submodel for the exacerbation severity and in the zero-inflated submodel, along with their 95% CIs. Given our parameterization of the model, a positive regression coefficient (AFT factor> 1 or OR > 1) corresponds to an increase in the mean of the distribution of between-exacerbation gap times represented by the rate submodel (or equivalently a decrease in the rate of exacerbations), an increase in the mean of the distribution of exacerbation durations (or equivalently a decrease in the rate of exacerbation termination), and an increase in the probability of severe exacerbation as the value of the covariate increases [30].

**Table 2:** AFT and OR estimates for the adjusted model with log-normal random effects

Covariate	Submodel			
	Between exacerbation	Duration	Severity	Zero-inflated
	AFT$^{\dagger }$ (95%CI)	AFT$^{\dagger }$ (95%CI)	OR (95%CI)	OR (95%CI)
Mepolizumab (75 mg)	1.60 (1.19, 2.16) *	0.97 (0.83, 1.13)	1.25 (0.65, 2.39)	2.11 (0.90, 4.92)
Mepolizumab (100 mg)	1.38 (0.99, 1.92)	0.95 (0.82, 1.09)	1.66 (0.70, 3.94)	1.59 (0.62, 4.05)
Mepolizumab (250 mg)	2.18 (1.44, 3.29) *	0.94 (0.76, 1.16)	1.14 (0.40, 3.27)	2.62 (0.95, 7.18)
Mepolizumab (750 mg)	2.02 (1.38, 2.96) *	0.85 (0.72, 1.00) *	1.30 (0.53, 3.17)	1.42 (0.47, 4.33)
Age (in decades)	1.09 (0.98, 1.22)	1.02 (0.97, 1.08)	0.79 (0.63, 1.00) *	1.00 (0.78, 1.28)
Female	1.08 (0.86, 1.36)	1.05 (0.93, 1.18)	1.20 (0.65, 2.23)	0.37 (0.19, 0.73) *
FEV1 (liter)	0.81 (0.40, 1.66)	1.11 (0.77, 1.59)	0.89 (0.19, 4.26)	6.22 (0.90, 43.1)
Nasal polyp	1.15 (0.83, 1.60)	1.00 (0.85, 1.19)	0.94 (0.37, 2.36)	1.27 (0.51, 3.15)
ACQ score	0.90 (0.80, 1.00)	1.01 (0.96, 1.06)	0.97 (0.71, 1.33)	0.70 (0.52, 0.95) *
BMI	1.07 (0.89, 1.28)	1.14 (1.04, 1.26) *	1.94 (1.26, 3.00) *	0.63 (0.33, 1.19)
FEV1 reversibility (percentage)	1.27 (0.86, 1.89)	0.90 (0.74, 1.09)	0.94 (0.38, 2.34)	0.26 (0.04, 1.85)
Baseline eosinophil	0.77 (0.55, 1.08)	1.08 (0.95, 1.24)	2.01 (0.53, 7.67)	1.49 (0.79, 2.83)
History of smoking	1.13 (0.87, 1.46)	0.96 (0.85, 1.08)	1.31 (0.69, 2.48)	0.47 (0.19, 1.13)
Duration of asthma (in decades)	0.98 (0.90, 1.06)	1.03 (0.99, 1.07)	1.08 (0.85, 1.37)	1.00 (0.80, 1.26)
Baseline SCS daily dose	0.96 (0.88, 1.05)	1.08 (1.03, 1.14) *	1.32 (1.06, 1.65) *	0.18 (0.04, 0.92) *
Black/Hispanic v. White	0.76 (0.55, 1.06)	0.85 (0.72, 1.00) *	4.93 (2.39, 10.17) *	1.30 (0.42, 4.00)
Baseline IgE	1.04 (0.95, 1.15)	0.96 (0.91, 1.02)	1.02 (0.75, 1.37)	0.64 (0.34, 1.22)
No. of exacerbations year prior to study	0.47 (0.34, 0.64) *	0.73 (0.63, 0.86) *	1.65 (0.87, 3.16)	0.33 (0.07, 1.44)
$N^{\ddagger }$	1.10 (1.02, 1.19) *	1.04 (1.00, 1.08) *	1.20 (1.00, 1.43)	-
$N_S^{\ddagger }$	0.73 (0.58, 0.93) *	0.99 (0.89, 1.10)	-	-

*Significant at 0.05 level AFT: accelerated failure time multiplicative factor ($= \exp (\beta )$); OR: odds ratio ($= \exp (\beta )$); FEV1: forced expiratory volume at one

second; ACQ: asthma quality of life questionnaire; SCS: systemic corticosteroid; BMI: body mass index

$^{\dagger }$: ln the AFT model, each regression coefficient, when exponentiated, yields the multiplicative factor by which a one unit change in the

covariate shortens/elongates the mean time to event

$^{\ddagger }$: Episode-specific covariates used in the model: *N* = number of previous exacerbations during follow-up; $N_S$ = number of previous

severe exacerbations during follow-up

With respect to the rate component, our analysis largely reproduced the findings of the DREAM and MENSA studies on the beneficial effects of mepolizumab on the between-exacerbation times: the 250 mg dose yielded the largest effect (AFT factor 2.18, 95%CI 1.44, 3.29). On the other hand, the joint model also allows detailed inference concerning both duration and severity, as well the interrelationships among all three dimensions of asthma exacerbations. The treatments seemed to be associated with modest decreases in exacerbation duration with some indication of a dose-response relationship. However, only the highest dose (750 mg) was significantly (at the conventional 0.05 significance level) associated with shorter exacerbation duration (AFT factor 0.85, 95% CI: 0.72, 1.00). No treatment effects were detected on exacerbation severity.

Table 3 reports the estimates of the shape parameters of the log-normal baseline hazard functions and the variance and correlation parameters of the random effects. Figure 3 shows the distribution of the estimated (empirical Bayes) random effects (panel A), their pairwise scatterplots (panel B), and marginal predicted mean outcomes (panel C) for patients who had at least one exacerbation. We excluded individuals with no exacerbations from this plot because the random effects for the duration and severity components cannot be estimated for this subgroup. Such plots can be used as a visual tool to assess the overall goodness of fit of the model as well as the necessity of including random effects in each submodel. According to the predicted values, there were substantial levels of between-individual variability (heterogeneity) in the burden of exacerbations: the mid-95% ranges of the marginal predicted means in the sample were: 147–840 days for the between exacerbation gap times, 11–27 days for the within exacerbation gap times, and 0.02$-$0.51 for the probabilities of an exacerbation being severe. Some of this heterogeneity can be attributed to between-individual variations in covariates. However, there was substantial unexplained variability in each of rate, duration and severity, as documented by the estimated random effect standard deviations of 0.78, 0.54, and 1.46, respectively, each with confidence intervals that are clearly removed from 0 (Table 3).

**Table 3:** Log-normal shape parameter and random effects parameter estimates

Parameter		Estimate	$95\%$ CI
Shape	Rate	1.22	(1.15, 1.29)
	Duration	0.62	(0.59, 0.65)
SD	Rate	0.78	(0.66, 0.90)
	Duration	0.54	(0.45, 0.63)
	Severity	1.46	(0.94, 1.97)
Correlation	Rate-Duration	0.40	(0.25, 0.55)
	Rate-Severity	0.47	(0.18, 0.76)
	Duration-Severity	0.47	(0.27, 0.68)

The correlation between the random effects for the rate and duration submodels was 0.40 (95% CI: 0.25, 0.55), indicating that, after controlling for observed characteristics, exacerbations in patients with more frequent exacerbations tended to be shorter. The corresponding correlation between the rate and severity submodels was 0.47 (95% CI: 0.18, 0.76), indicating that more frequent exacerbators tended to have fewer severe exacerbations. The duration and severity submodels were also positively correlated (0.47, 95% CI: 0.27, 0.68), indicating that severe exacerbations tended to be longer in duration than non-severe ones. Section 3 of the Additional file 1 illustrates the extent to which the model adequately reflects the correlations between the outcomes. In addition, Section 3.3 of the Additional file 1 presents the results of a sensitivity analysis based on multiple imputation of missing data, which generated similar results to the main analysis (which excluded observations with missing values).

## Discussion

We developed and implemented a parametric multi-component model to jointly characterize the three important dimensions of exacerbations or flare-ups present in many chronic diseases: their rate, duration, and severity. Our model can describe the relationships between these three aspects of the natural history of exacerbations, can quantify the between-individual variability (heterogeneity) in each of these aspects, and can elucidate the effect of exposures (e.g., intervention in a clinical trial) on each of these dimensions simultaneously.

As a case study, we implemented our method using pooled data from two large asthma trials. Some of the novel findings from the application of this unified modeling approach were the potential effect of the treatment on the duration of exacerbations, which was not evaluated in the original studies, and the elucidation of dependencies among the rate, duration, and severity of exacerbations. We could demonstrate that, after controling for observable characteristics, exacerbations among more frequent exacerbators tended to be shorter and less likely to be severe. This might reflect a ‘threshold’ effect: that different patients might have different thresholds in registering a period of intensified disease activity as an exacerbation; thus, those with lower thresholds will tend to report more exacerbations, but a lower fraction of such exacerbations would be severe enough that would require urgent or inpatient care.

To the best of our knowledge, this is the first study that jointly characterizes the three important aspects of exacerbations. Most studies to date have only modeled the rate of a complete healthy or sick episode [24, 33–36], while a few have jointly modeled rate and severity of the episodes but did not consider their duration [6]. In addition to providing novel insights into the natural history of such diseases, the proposed approach can mitigate some of the biases that can arise from not modeling these components jointly. As an example, [18] showed that ignoring the durations can lead to serious bias in estimating the rate of COPD exacerbations. Moreover, the model addresses the issue of the excessive presence of individuals without any exacerbations during follow-up (zero-inflation), which has been frequently discussed in the literature [13–15]. Importantly, the proposed framework can be implemented with standard statistical software; to enhance its accessibility, an annotated SAS macro that implements the model in a generic fashion, along with a manual and a simulated dataset, is available at our website (http://resp.core.ubc.ca/software/RDSmodel).

Our study also has limitations. We used fully parametric models for the time to start/end of exacerbations. While such models accommodate straightforward estimation of random effects and facilitate outcome prediction, parametric modeling inherently involves stronger assumptions on the shape of the hazard function compared with the semi-parametric methods such as the Cox proportional hazards model. One can employ more flexible approaches (such as splines) to model time-to-event data if the common parametric distributions do not perform well in an application (this was not needed in our case study) [37–40]. Further, we dichotomized exacerbation severity as moderate (requiring outpatient care) versus severe (requiring emergency department visit or hospital admission). One can easily extend the current model to incorporate severity as an ordinal categorical outcome by replacing the severity submodel with a multinomial or ordinal model. Overall, as our focus was on proposing a novel methodology rather than providing definitive clinical results, we have made simplifying assumptions (removal of outlying variables, dichotomization of outcome), but in general this framework is flexible for further expansion to accommodate such features.

Characterizing between-individual variability (heterogeneity) in different dimensions of exacerbations, through implementing random effects, is an important feature of the proposed approach. In addition, such a joint model can potentially facilitate prediction of future burden of exacerbations, including their rate, duration, and severity in tandem, albeit the predictive performance of such a modeling framework needs to be investigated in future studies [41]. It is worth mentioning that incorporating random effects in all components of the model may require substantial computation, particularly when the number of covariates present in each submodel is large, and convergence issues may arise (of note, the model employed in the case study required 30 min on a typical personal computer with 2.3 GHz in CPU and 16GB of RAM). Evaluating the feasibility of this approach with larger datasets and with the inclusion of more covariates should be studied. Additionally, incorporating other distributions for the random effect terms or more generally treating them non-parametrically [42] can be pursued in future studies. The approach could be further enhanced by extending the framework to incorporate competing risks and a more generally applicable approach for left-truncated outcomes. Although not required for our case study, these enhancements may be important in other contexts. Modeling such recurrent episodes data by using multi-state models or approaches to model point processes could be some alternative models that can be considered as further extensions to the present work.

In summary, the burden of exacerbations, a shared feature of many chronic diseases, is not manifested only in their frequency, but also in their duration and severity. Through joint modeling of these aspects of exacerbations, the proposed framework has the potential to improve our understanding of the natural history of episodic conditions and their overall burden. In addition, it enables the exploration of different aspects of how treatments can impact the burden of episodic diseases, ultimately improving our ability to quantify the benefit of treatments given each patient’s unique characteristics.

## Supplementary Information

**Additional file 1:** Peer Review Reports. The Additional file Includes the likelihood and some additional outcomes of the fitted model in our case study.

## Data Availability

The datasets used in this study are not publicly available. We obtained the access to these data from the Clinical Study Data Request team (research proposal M17-00036). Researchers needing to access these datasets should submit a data request to the Clinical Study Data Request team (www.clinicalstudydatarequest.com). They will be contacted by the data sharing committee to discuss the next steps for access to the data files. All the other materials including the SAS macro and manual are available on our RESP lab website (http://resp.core.ubc.ca/software/RDSModel). The analysis code is available from https://github.com/resplab/papercode/tree/main/RDSmodel.

## Abbreviations

ACQ: Asthma control questionnaire
AFT: Accelerated failure time
AIC: Akaike Information Criterion
BMI: Body mass index
CI: Confidence interval
COPD: Chronic obstructive pulmonary disease
ED: Emergency department
FEV1: Forced expiratory volume at one second
OR: Odds ratio
SCS: Systemic corticosteroid
SD: Standard deviation
