# Supplementary material for: A parametric model to jointly characterize rate, duration, and severity of exacerbations in episodic diseases
Source: BMC Med Inform Decis Mak. 2023 Jan 12;23:6. doi: 10.1186/s12911-022-02080-5 (PMC9837953; doi:10.1186/s12911-022-02080-5)
Supplement: Supplementary file 1 — Additional file 1: Peer Review Reports. The Additional file Includes the likelihood and some additional outcomes of the fitted model in our case study. [file 12911_2022_2080_MOESM1_ESM.pdf]

Supplementary materials for "A parametric model to jointly characterize rate, duration, and severity of exacerbations in episodic diseases"

By A. Safari, J. Petkau, J. M. FitzGerald, and M. Sadatsafavi

1 Collapsing treatment arms across studies

As a preliminary step, we checked the comparability of the covariates across studies to see whether it is reasonable to collapse the data on the two treatment arms that are common to the two studies (placebo and 75 mg Mepolizumab). Table 1 suggests some differences so we proceeded to more formal assessment via model comparisons, incorporating study ID and its interactions with the common treatment arms as extra predictors in all the submodels. These extra predictors had little impact on the model goodness of fit, as assessed both by model AIC and significance level (p-value>0.1 for all these extra predictors). The results indicate that collapsing common treatment arms across studies is reasonable.

Supplementary Table 1. Descriptive statistics of the predictors and outcomes before and after collapsing the treatment arms

Predictors	Placebo * (n=323)	Placebo DREAM (n=153)	Placebo MENSA (n=170)	75mg Mepo * (n=317)	75mg Mepo DREAM (n=149)	75mg Mepo MENSA (n=168)
Female	193(60%)	96 (63%)	97 (57%)	193 (61%)	101 (67%)	92 (55%)
Age (years)	47.7 (13.2)	46.3 (11.3)	48.9 (14.6)	49.8 (13.0)	50.1 (11.0)	49.6 (14.6)
Body Mass Index (kg/m2)	28.2 (5.8)	28.3 (6.1)	28.1 (5.6)	28.0 (5.9)	28.4 (6.0)	27.6 (5.7)
Duration of asthma (years)	18.8 (14.3)	18.1 (13.6)	19.4 (14.8)	19.3 (13.9)	18.9 (14.0)	19.7 (13.8)
Maintenance daily dose of oral corticosteroids (mg)	3.5 (8.2)	4.6 (9.9)	2.5 (6.0)	3.7 (8.9)	5.1 (10.9)	2.5 (6.5)
Nasal polyps	47 (14%)	16 (11%)	31 (18%)	40 (13%)	11 (7%)	29 (17%)
Percentage of predicted pre-bronchodilator FEV1	61.1 (16.9)	58.7 (14.9)	63.1 (18.4)	60.2 (17.5)	59.4 (15.9)	61.0 (18.8)
FEV1 Reversibility (%)	26.7 (22.4)	26.6 (24.5)	26.7 (20.3)	26.2 (20.2)	25.1 (21.2)	27.3 (19.4)
Score on asthma control questionnaire	2.3 (1.3)	2.5 (1.1)	2.2 (1.1)	2.2 (1.1)	2.2 (1.0)	2.1 (1.1)
Blood eosinophil count ($\times 10^9/L$)	0.44 (0.42)	0.42 (0.37)	0.47 (0.46)	0.40 (0.38)	0.37 (0.35)	0.43 (0.41)
IgE (U/ml)	435.3 (850)	457.0 (687)	415.8 (975)	548.3 (1318)	427.9 (689)	655.0 (1685)
Ethnicity (Black/Hispanic)	31 (10%)	16 (10%)	15 (9%)	32 (10%)	14 (9%)	18 (10%)
History of smoking	67 (21%)	33 (22%)	34 (20%)	74 (24%)	29 (20%)	45 (27%)
Severe exacerbations in year prior to study	3.7 (3.3)	3.7 (3.8)	3.6 (2.8)	3.6 (2.7)	3.7 (3.2)	3.5 (2.2)
Exacerbations requiring admission in year prior to study	198 (17%)	58 (10%)	140 (22%)	153 (13%)	44 (10%)	109 (18%)
Outcomes						
Number of clinically significant exacerbations (Rate)	468 (1.94)	283 (2.05)	185 (1.81)	251 (1.05)	152 (1.10)	99 (0.98)
Number of exacerbations requiring ER or hospitalization (Rate)	68 (0.28)	40 (0.29)	28 (0.27)	39 (0.16)	17 (0.12)	22 (0.22)

* Pooled data from both studies

2 The likelihood

We begin by describing the contribution of patient i to the likelihood conditional on the random effects for our case study. One can modify these contributions to the settings of other applications in a straightforward fashion. If patient i has

no exacerbations over the follow-up period, its contribution to the conditional likelihood is

$$\mathcal{L}_i\left(\boldsymbol{\beta}|\boldsymbol{Z_i}, \boldsymbol{X}_{i,1}\right) = \pi_i + (1-\pi_i)\exp\left(-\int_0^{T_i} h_{i,j,B}(t)dt\right)$$

where $\boldsymbol{\beta} = (\boldsymbol{\beta}_B^T, \boldsymbol{\beta}_W^T, \boldsymbol{\beta}_S^T, \boldsymbol{\beta}_{ZI}^T)^T$. For patients with at least one exacerbation over the follow-up period, let $a_i = 1$ if patient i ends the nominal follow-up period during an exacerbation (in this case, $T_i = v_{i,M_i}$, the time of the end of this exacerbation) and $a_i = 0$ otherwise. The contribution of patient i to the conditional likelihood is

$$\begin{aligned}\mathcal{L}_i(\boldsymbol{\beta}|\boldsymbol{Z_i}, \boldsymbol{X_i}) &= (1-\pi_i)\times \\ &\prod_{j=1}^{M_i}\left[\exp\left(-\int_{v_{i,j-1}}^{u_{i,j}} h_{i,j,B}(t)dt\right) h_{i,j,B}(u_{i,j}) p_{i,j}^{s_{i,j}}(1-p_{i,j})^{1-s_{i,j}}\times\right. \\ &\qquad \left.\exp\left(-\int_{u_{i,j}}^{v_{i,j}} h_{i,j,W}(t)dt\right) h_{i,j,W}(v_{i,j})\right]\times \\ &\left[\exp\left(-\int_{v_{i,M_i}}^{T_i} h_{i,M_i+1,B}(t)dt\right)\right]^{1-a_i}\end{aligned}$$

where $\boldsymbol{X_i} = (\boldsymbol{X}_{\boldsymbol{i,1}}^T, \ldots, \boldsymbol{X}_{\boldsymbol{i,M_i+1}}^T)^T$. Note that if the non-susceptible component is not required in an application, one can set $\pi_i \equiv 0$.

To obtain the full likelihood, we integrate the contribution of patient i over the multivariate normal distribution of the three random effects and take the product of these (marginal) likelihood contributions across all patients.

3 Additional Results

3.1 AIC comparison

Table 2 shows the AIC results of four different baseline hazard functions when these are used for both the rate and duration components of the model (the AIC results were similar for other combinations of these distributions for the rate and duration submodels). The log-normal distribution for both rate and duration submodels has the lowest AIC for the case study.

Supplementary Table 2. The AIC results of the full model for different AFT models

Model	AIC
Log-normal	285.9
Log-logistic	305.1
Weibull	502.6
Exponential	780.3

3.2 Correlation between outcomes

We can use the estimated random effect values (along with other estimated regression coefficients) to predict all the outcomes (between and within exacerbation times, and severity statuses) for each patient. We can then use these predicted values to estimate the correlations between each pair of outcomes based on our fitted model. The average of such correlations over multiple predicted

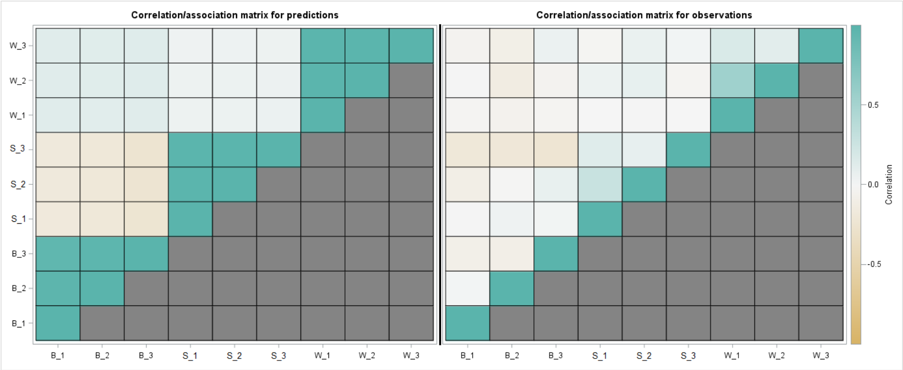

Supplementary Figure 1. Correlation heatmap plot of the first three episodes among the predictions (left) and observations (right)

data sets estimates the true correlation. We generated 100 predicted data sets for patients who had at least three exacerbations during the follow-up time. Figure 1 shows the heatmap plots for the correlations between the predicted (left panel) and observed outcomes (right panel) for the first three episodes. We use the Pearson correlation coefficients for pairs with numeric outcomes (between and within exacerbation times) and Kendall's tau coefficient (association) otherwise. The pattern of the two sets of correlations are similar except the magnitude of correlations in the predictions are slightly larger than those of the observations. The results indicate that the assumed correlation structure in the model is reasonable.

3.3 Sensitivity Analysis

To assess the impact of excluding patients with missing values in their covariates, we carried out a sensitivity analysis. We used multiple imputation to generate 5 different datasets with imputed values for the missing values, and interpreted the treatment effect (main covariates of interest) as well as the random effect parameter estimates in terms of their sensitivity to the use of complete cases versus imputed data. As the Table 3 reports, the estimates from the two analyses were.

Supplementary Table 3. The treatment effect and random effect parameter estimates based on both the complete cases and the imputed data

Data	Covariate/RE	Submodel				RE Correlation
		Between exacerbation AFT (SD)	Duration AFT (SD)	Severity OR (SD)	Zero-inflated OR (SD)	
Complete cases	Mepolizumab (75mg)	1.60 (1.13)	0.97 (0.07)	1.25 (0.43)	2.11 (0.88)	
	Mepolizumab (100mg)	1.38 (0.23)	0.95 (0.07)	1.66 (0.79)	1.59 (0.74)	
	Mepolizumab (250mg)	2.18 (0.44)	0.94 (0.10)	1.14 (0.61)	2.62 (1.30)	
	Mepolizumab (750mg)	2.02 (0.38)	0.85 (0.07)	1.30 (0.63)	1.42 (0.79)	
	Rate-Duration					0.40 (0.07)
	Rate-Severity					0.47 (0.14)
	Duration-Severity					0.47 (0.10)
Imputed data	Mepolizumab (75mg)	1.61 (0.23)	0.96 (0.07)	1.29 (0.33)	2.09 (0.58)	
	Mepolizumab (100mg)	1.39 (0.23)	0.95 (0.08)	1.79 (0.51)	1.57 (0.51)	
	Mepolizumab (250mg)	2.22 (0.47)	0.93 (0.09)	1.15 (0.45)	2.56 (0.96)	
	Mepolizumab (750mg)	2.05 (0.35)	0.85 (0.07)	1.38 (0.40)	1.40 (0.48)	
	Rate-Duration					0.40 (0.07)
	Rate-Severity					0.46 (0.15)
	Duration-Severity					0.47 (0.11)
